# Rates of SARS-CoV-2 Breakthrough Infection or Severe COVID-19 and Associated Risk Factors After Primary and Booster Vaccination Against COVID-19 in the Netherlands

**DOI:** 10.3390/vaccines13060564

**Published:** 2025-05-26

**Authors:** Jesse M. van den Berg, Marieke T. Blom, Jetty A. Overbeek, Sharon Remmelzwaal, Ron M. C. Herings, Petra J. M. Elders

**Affiliations:** 1 PHARMO Institute for Drug Outcomes Research, 3528 AE Utrecht, The Netherlands; 2 Department of General Practice, Amsterdam UMC Location Vrije Universiteit Amsterdam, 1081 HV Amsterdam, The Netherlands; 3 Amsterdam Public Health Research Institute, Health Behaviors and Chronic Diseases, 1105 HV Amsterdam, The Netherlands; 4 Department of Epidemiology and Data Science, Amsterdam UMC Location Vrije Universiteit Amsterdam, 1081 HV Amsterdam, The Netherlands

**Keywords:** COVID-19, SARS-CoV-2, coronavirus, vaccination, vaccine effectiveness, risk factors, comorbidities

## Abstract

**Background:** The effectiveness of COVID-19 vaccines appears to decline rapidly over time due to waning immunity and immune evasion by emerging variants of concern, and may be reduced in high-risk populations. We aimed to evaluate the rates of SARS-CoV-2 breakthrough infection or severe COVID-19, both in individuals who had completed their primary COVID-19 vaccination, and in those who had received their first booster vaccination. Specifically, we aimed to evaluate whether persons with certain risk factors, such as age, gender, socioeconomic status (SES), and specified comorbidities have an increased risk of either breakthrough infection or severe COVID-19, compared to those without the respective risk factors. **Methods:** Data on COVID-19 vaccinations, infections, hospitalizations, and deaths were collected from the PHARMO Data Network, consisting of health records from Dutch residents. Two cohorts were established: (1) all persons who have completed their primary COVID-19 vaccination regimen, and (2) those who have received their first booster vaccination. The outcomes were SARS-CoV-2 breakthrough infection, and severe COVID-19, defined as either hospitalization or death following SARS-CoV-2 infection. Incidence rates of these outcomes were calculated in both cohorts. The adjusted incidence rate ratios of these outcomes in persons with certain risk factors were calculated, using generalized linear models with a Poisson distribution. **Results:** In 2021, a total of 1,090,567 individuals received either two doses of BNT162b2, AZD1222, or mRNA-1273, or one dose of Ad26.COV2.S and were included in the primary vaccination cohort, of which 344,153 (31.6%) received a booster vaccination. Overall incidence rates of SARS-CoV-2 breakthrough infection and severe COVID-19 after primary vaccination were 29.9 and 3.1 per 1000 person-years, respectively, and after booster vaccination were 256.4 and 2.3, respectively. Male gender, older age, lower SES, history of COVID-19, and recent hospitalization were factors associated with a lower risk of breakthrough infection after primary vaccination, and a higher risk of severe COVID-19. The risk of severe COVID-19 after primary vaccination was increased in persons with several comorbidities, compared to those without, and remained elevated after booster vaccination in persons with diabetes or lung disease. **Conclusions:** Our study emphasizes the crucial role of boosters in reducing breakthrough infections, particularly in high-risk populations. The varied impact on severe outcomes in individuals with comorbidities underscores the need for ongoing surveillance and tailored vaccination strategies.

## 1. Introduction

Since the introduction of COVID-19 vaccines in 2020, starting with the approval of BNT162b2 in December 2020, these have shown high efficacy in preventing severe COVID-19, but their effectiveness in preventing SARS-CoV-2 infection is comparatively low [1,2,3,4]. The effectiveness of COVID-19 vaccines appears to decline over time due to waning immunity and immune evasion by emerging variants of concern, and may be further reduced in high-risk populations [5,6,7]. Consequently, repeat vaccinations were necessary as the COVID-19 pandemic continued, as has been the policy in the Netherlands. Although risk factors for severe COVID-19 are well known, it remains unclear which populations would benefit most from repeat vaccination [8]. More research is needed to assess whether the effectiveness of COVID-19 vaccination is affected by potential risk-increasing factors, such as the presence of certain comorbidities. Recent research has shown that effectiveness may be reduced in high-risk populations, such as older persons, or in those with comorbidities such as diabetes mellitus, cardiovascular disease, neurological disease, lung disease, malignancy, kidney disease, immune deficiency, and obesity [9,10,11,12,13,14,15]. However, there is still a paucity of evidence regarding the effectiveness of COVID-19 vaccination in persons with these specific comorbidities, compared to those without. More information on this subject may be important for COVID-19 vaccination policy to prioritize and consider additional vaccinations in these high-risk populations.

The effectiveness of vaccination is often assessed by comparing the rate of the outcome in vaccinated persons with the rate of the outcome in unvaccinated persons [16]. Ideally, this is performed using a test-negative design, but can also be done using a cohort or case-control study [17,18,19,20]. However, the effectiveness of COVID-19 vaccination can also be studied in a different way. Instead of comparing outcomes in vaccinated and unvaccinated persons, the risk of infection or severe illness can be determined in vaccinated persons only. In particular, comparing the rates of these outcomes in persons with and without certain risk factors can provide information on the effectiveness of vaccination in these specific populations. Among vaccinated individuals, infection occurring despite vaccination is referred to as a breakthrough infection, which is defined by the WHO as the detection of SARS-CoV-2 RNA or antigen in a respiratory specimen collected from a person ≥14 days after they have completed all recommended doses of the vaccine series [21].

Our aim was to evaluate the rates of SARS-CoV-2 breakthrough infection or severe COVID-19, both in individuals who have completed their primary COVID-19 vaccination, and among those who have received their first booster vaccination. Specifically, we aimed to evaluate whether persons having certain risk factors, such as age, gender, socioeconomic status, and pre-specified comorbidities, have an increased risk of either breakthrough infection or severe COVID-19, compared to persons without these respective risk factors. This approach will contribute to a deeper understanding of the effectiveness of the COVID-19 vaccination in different subgroups of the population and, consequently, should help to inform targeted vaccination strategies and public health interventions.

## 2. Methods

### 2.1. Data Setting

We performed a population-based retrospective cohort study using Electronic Health Records (EHR) from PHARMO’s Dutch General Practitioner (GP) data, which hold records of all non-institutionalized patients under the care of GPs. The GP data cover a catchment area of approximately 3.2 million inhabitants (~20% of the Dutch population). The EHR data include information on prescriptions, diagnoses and symptoms, laboratory test results and referrals to specialists. The hospital data consist of datasets that contain detailed data on hospital admissions, outpatient consultations and high-cost medications; for the current study, only information on admissions was used. COVID-19 infection and vaccination status were mainly identified as reported by the municipal public health services (GGD) to the GP, by GP vaccination records, or by vaccination reports from hospitals or health care facilities. More information on the PHARMO Data Network has been published elsewhere [22,23]. The study was approved by the Institutional Review Board of Stichting Informatievoorziening voor Zorg en Onderzoek (STIZON, reference number CC2025-09, 13 February 2025).

### 2.2. Study Population

**Figure 1:**
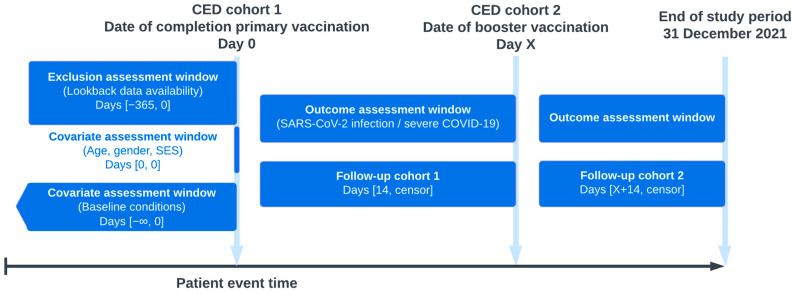
Study diagram with description of cohorts, including assessment windows of exclusions, covariates, and outcomes for both the primary vaccination cohort and the booster vaccination cohort. Persons with ≤1 dose were excluded (except for Ad26.COV2.S vaccine). Censoring of cohort 1 occurred at either SARS-CoV-2 breakthrough infection, COVID-19 related hospitalization, death, end of follow-up, or date of booster vaccination, whichever came first. Censoring of cohort 2 occurred at either SARS-CoV-2 breakthrough infection, COVID-19 related hospitalization, end of follow-up, death, or end of study period, whichever came first. CED, cohort entry date; SES, socioeconomic status.

From the GP data we included all individuals aged 18 years or older who were active in the GP data during the study period, and were eligible to have information on hospital data. Of these persons, we included those who were vaccinated with BNT162b2 (Comirnaty; Pfizer-BioNTech, New York, NY, USA), AZD1222 (Vaxzevria; AstraZeneca, Cambridge, UK), mRNA-1273 (Spikevax; Moderna, Cambridge, MA, USA), or Ad26.COV2.S (Jcovden; Janssen, Leiden, The Netherlands), as registered in the GP data during the study period from 1 January 2021 to 31 December 2021, when the nationwide rollout of COVID-19 vaccines was initiated in the Netherlands. We excluded individuals who received only one vaccination (except for Ad26.COV2.S, in which one dose was deemed sufficient for complete vaccination) and individuals with less than 1 year of data availability before the date of the second vaccine dose (Figure 1). Two cohorts were created: (1) primary vaccination cohort: persons who had received two doses of a COVID-19 vaccine, or one dose when receiving Ad26.COV2.S; and (2) booster vaccination cohort: persons who had received a (first) booster vaccination. The cohort entry date (CED) for cohort 1 was the date of completing their primary vaccination and the CED for cohort 2 was the date of the booster vaccination. Follow-up was from 14 days after CED 1 for cohort 1 and 14 days after CED 2 for cohort 2. Censoring of cohort 1 occurred at either a SARS-CoV-2 breakthrough infection, COVID-19-related hospitalization, death, end of follow-up, or date of booster vaccination, whichever came first. Censoring of cohort 2 occurred at either a SARS-CoV-2 breakthrough infection, COVID-19 related hospitalization, end of follow-up, death, or end of study period, whichever came first (Figure 1).

### 2.3. Outcomes

Outcomes are (1) SARS-CoV-2 breakthrough infection after vaccination, defined as either a positive SARS-CoV-2 PCR test or diagnosis of COVID-19 (i.e., using an algorithm that includes either ICPC-1 code R83.03 or a free text in medical records mentioning COVID-19, and subsequently excludes irrelevant records), from 14 days after vaccination onwards, aligned with the WHO definition [21], and (2) severe COVID-19, defined as either COVID-19-related hospitalization or all-cause mortality within 28 days of a SARS-CoV-2 infection [24].

### 2.4. Characteristics and Covariates

Patient characteristics were defined at cohort entry date 1 (CED 1), with a look-back window of at least 1 year, and include age, gender, socioeconomic status (SES), SARS-CoV-2 infection prior to baseline, hospitalization in the 28 days before vaccination, and smoking status. The presence of the following comorbidities was examined: diabetes mellitus, cardiovascular disease, neurological disease, lung disease, malignancy, kidney disease, immune deficiency, and obesity. Comorbidities were assumed to be present if recorded in the electronic health records, and assumed to be absent if not recorded anywhere in the patient’s records. All covariates, including their definitions, are described in more detail in Supplementary Table S1.

### 2.5. Statistical Analysis

Descriptive statistics were used to summarize baseline characteristics as proportions of characteristic categories. Incidence rates (IRs) of severe COVID-19 and SARS-CoV-2 breakthrough infection were calculated as the number of outcomes per 1000 person-years, for both cohort 1 and cohort 2. We used generalized linear models with a Poisson distribution, with a log link function, with person-time offset for time at risk, to calculate Poisson adjusted incidence rate ratios (IRRs) for severe COVID-19 and SARS-CoV-2 breakthrough infection. We used robust standard errors to account for mild overdispersion. We calculated these IRRs after primary vaccination and after booster vaccination. Also, we calculated both unadjusted and adjusted IRRs including the following covariates: age, gender, SES, SARS-CoV-2 infection before the first vaccine dose, hospitalization in the 28 days before vaccination, smoking status, and timing of vaccination (quarter). Models of IRRs for age, gender, SES, history of SARS-CoV-2 infection, recent hospitalization, smoking status, and timing of vaccination were all univariate models. Models of IRRs for comorbidities were constructed using multiple models. Model 1 adjusted for age and gender. Model 2 extended model 1 by additionally adjusting for SES, history of SARS-CoV-2 infection, recent hospitalization, smoking status, and timing of vaccination. Model 3 extended model 2 by adjusting for all other comorbidities (excluding the one being investigated). All statistical tests were two-tailed with a 5% significance level. Several sensitivity analyses were performed, including IRRs for severe COVID-19 and SARS-CoV-2 breakthrough infection, after a primary vaccination and after a booster vaccination, but separately for BNT162b2, AZD1222, mRNA-1273, and Ad26.COV2.S. Additionally, IRRs for severe COVID-19 were recalculated using all-cause mortality within 90 days of a SARS-CoV-2 infection instead of 28 days. Several variables were missing for a relatively large proportion of the study population, which is mainly attributable to the availability of vaccination information from almost all patients registered in the GP data, while a considerable number of these patients will not have visited their GP for medical reasons. This could have caused certain variables to be more likely to not be recorded (e.g., smoking status, or BMI), as opposed to persons who do regularly visit their GP, who may generally be less healthy. Absence of data can therefore be informative. Accordingly, missing categorical variables (e.g., smoking status) were handled by introducing “unknown” as a separate category in the regression models. The vaccine manufacturer was abstracted from the free text or vaccine batch number in the electronic health records, and could be missing due to registration issues. Vaccinated individuals with an unknown vaccine manufacturer were included in the main analyses, but excluded from sensitivity analyses. All statistical analyses were conducted using R (version 4.3.1), with generalized linear models performed using the stats (version 4.3.1) and MASS (version 7.3-60) packages.

## 3. Results

From 1 January 2021 until 31 December 2021, a total of 1,090,567 individuals received either two doses of BNT162b2, AZD1222, or mRNA-1273, or one dose of Ad26.COV2.S and were thus included in cohort 1. Of these persons, 344,153 (31.6%) individuals received a booster vaccination during the study period and were subsequently included in cohort 2.

Mean age in cohort 1 was 49.8 (SD 21.5) years, and 538,787 (49.4%) persons were female. Most persons received BNT162b2 (792,349; 72.7%), followed by AZD1222 (103,189; 9.5%), mRNA-1273 (78,755; 7.2%), Ad26.COV2.S (46,972; 4.3%), or an unknown manufacturer (69,302; 6.4%) (Supplementary Tables S2 and S4). Overall incidence rates of SARS-CoV-2 breakthrough infection or severe COVID-19 after primary vaccination were 29.9 and 3.1 respectively, per 1000 person-years.

Mean age in cohort 2 was 68.4 (SD 12.8) years, and 163,339 (47.5%) persons were female. More than half received mRNA-1273 (191,222; 55.6%); the others received BNT162b2 (67,958; 19.7%), or an unknown manufacturer (84,973; 24.7%) (Supplementary Tables S3 and S5). Overall incidence rates of SARS-CoV-2 breakthrough infection or severe COVID-19 after booster vaccination were 256.4 and 2.3, respectively, per 1000 person-years (Table 1).

Table 2 shows the incidence rate ratios (IRRs) for SARS-CoV-2 breakthrough infection and severe COVID-19 after a primary vaccination, and IRRs after a booster vaccination. Men had a lower risk of infection than women after a primary vaccination (IRR 0.84; 95% CI 0.78–0.89). The risk was comparable with women after a booster vaccination (IRR 0.98; 0.94–1.03). Men had a higher risk of severe COVID-19 than women after both a primary (IRR 1.48; 1.25–1.75) and a booster (IRR 2.00; 1.42–2.81) vaccination. A lower risk of infection was seen in persons aged 60–69 years (IRR 0.78; 0.71–0.85), those aged 70–79 years (IRR 0.78; 0.72–0.86) and in those aged over 80 years (IRR 0.83; 0.75–0.92) compared with persons aged 18–59. A booster vaccination further reduced this risk (IRR 0.62, 0,49 and 0.36, respectively). Despite this lower risk of infection, a higher risk of severe COVID-19 was seen in persons aged 60–69 (IRR 2.94; 2.18–3.97), aged 70–79 (IRR 4.96; 3.80–6.47), and over 80 (IRR 9.90; 7.68–12.8), compared with persons aged 18–59. A booster vaccination reduced these risks of severe COVID-19 in persons aged 60–69 (IRR 1.56; 0.91–2.69), aged 70–79 (IRR 1.23; 0.71–2.13), and in those over 80 (IRR 1.91; 1.11–3.31). High socioeconomic status (SES) was associated with an increased risk of infection compared with those with a low SES after both a primary (IRR 1.20; 1.11–1.31) and a booster (IRR 1.33; 1.26–1.41) vaccination. While those with a high SES had a lower risk of severe COVID-19 after a primary vaccination (IRR 0.67; 0.54–0.83), no significant differences were observed after a booster vaccination. A history of COVID-19 was associated with a significantly reduced risk of infection after both a primary (IRR 0.30; 0.22–0.41) and a booster (IRR 0.38; 0.32–0.45) vaccination. Despite this reduced risk of infection, their risk of severe COVID-19 was greatly increased after a primary vaccination (IRR 4.86; 3.85–6.15), especially after a booster vaccination (IRR 14.8; 10.5–20.7), compared with those with no history of COVID-19. Recent hospitalization was not associated with a risk of infection, but was associated with a much higher risk of severe COVID-19, after both a primary (IRR 20.7; 14.9–28.8) and a booster (IRR 19.3; 10.5–35.3) vaccination.

**Figure 2:**
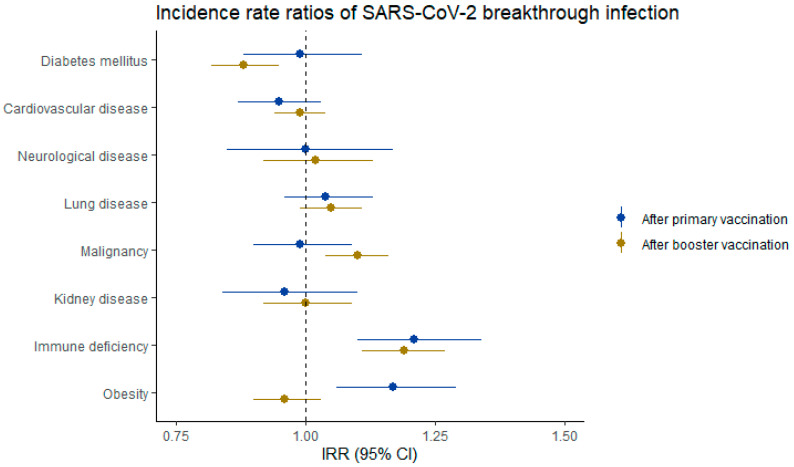
Forest plot of incidence rate ratios (IRRs) of SARS-CoV-2 breakthrough infection after primary vaccination, and after booster vaccination. Primary vaccination is defined as having received two doses of BNT162b2, mRNA-1273 or AZD1222, or one dose of Ad26.COV2.S. Booster vaccination is defined as having received a third dose, being either BNT162b2 or mRNA-1273. Corresponding IRRs are reported in Table 2.

**Figure 3:**
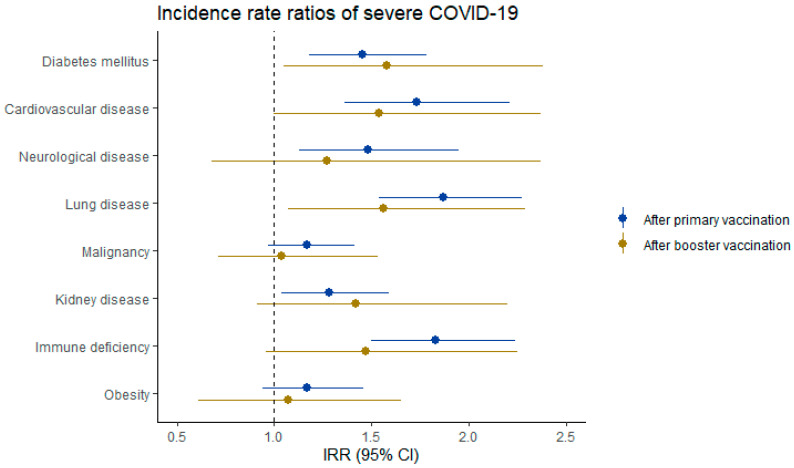
Forest plot of incidence rate ratios (IRRs) of severe COVID-19 after primary vaccination, and after booster vaccination. Primary vaccination is defined as having received two doses of BNT162b2, mRNA-1273 or AZD1222, or one dose of Ad26.COV2.S. Booster vaccination is defined as having received a third dose, being either BNT162b2 or mRNA-1273. Corresponding IRRs are reported in Table 2.

IRRs were also calculated for persons with diabetes mellitus, cardiovascular disease, neurological disease, lung disease, malignancy, kidney disease, immune deficiency, or obesity, compared with persons without these respective conditions. Risk of SARS-CoV-2 breakthrough infection after a primary vaccination was increased in persons with immune deficiency (IRR 1.21; 1.10–1.34) or obesity (IRR 1.17; 1.06–1.29); the IRRs for the other comorbidities were neither increased nor decreased. Risk of infection after a booster vaccination was decreased in persons with diabetes (IRR 0.88; 0.82–0.95) and increased in persons with malignancy (IRR 1.10; 1.04–1.16) or immune deficiency (IRR 1.19; 1.11–1.27). Risk of severe COVID-19 after a primary vaccination remained high in persons with comorbidities, and this risk was most increased in persons with lung disease (IRR 1.87; 1.54–2.27), immune deficiency (IRR 1.83; 1.50–2.24), or cardiovascular disease (IRR 1.73; 1.36–2.21). Risk of severe COVID-19 decreased after a booster vaccination for almost all comorbidities, but remained elevated in persons with diabetes (IRR 1.58; 1.05–2.38) or lung disease (IRR 1.56; 1.07–2.29), compared with those without. Forest plots of IRRs of breakthrough infection and severe COVID-19 in persons with and without investigated comorbidities are presented in Figure 2 and Figure 3, respectively. IRRs for comorbidities were estimated using multiple models (Supplementary Tables S7 and S8). After adjustment for additional covariates in model 2 and model 3, the IRRs generally shifted closer to 1 compared to model 1.

Sensitivity analyses were performed in which IRRs for both SARS-CoV-2 breakthrough infection and severe COVID are calculated for separate vaccine manufacturers, after a primary vaccination (Supplementary Tables S2 and S3) and after a booster vaccination (Supplementary Tables S4 and S5). No large differences from the results in Table 2 were observed. The results in the main analysis were mainly driven by the most administered vaccine brand for the primary vaccination cohort and the booster vaccination cohort, being BNT162b2 and mRNA-1273, respectively. Of note, IRRs for severe COVID-19 after a primary vaccination with Ad26.COV2.S could not be calculated, as models did not converge due to few or no outcomes per risk factor in this population. Additionally, IRRs for severe COVID-19 after a primary vaccination and after a booster vaccination were calculated considering all-cause mortality within 90 days of a SARS-CoV-2 infection instead of 28 days (Supplementary Table S6). Results of this sensitivity analysis are very similar to the results of the main analysis, with only slightly higher IRRs in persons with comorbidities.

## 4. Discussion

We evaluated the incidence rates of SARS-CoV-2 breakthrough infection and the rates of severe COVID-19 after both a primary vaccination and after a booster vaccination. The incidence rate of breakthrough infection during the study period was 29.9 events per 1000 person-years after primary vaccination, and 256.4 events per 1000 person-years after a booster vaccination. This increase can be explained by the higher incidence rates of COVID-19 in the Netherlands during the period after a booster vaccination compared to the period after a primary vaccination. Male gender, older age, lower SES, a history of COVID-19, a recent hospitalization, and a later date of vaccination in 2021 were factors associated with a lower risk of breakthrough infection after a primary vaccination.

Incidence rates of severe COVID-19 were 3.1 and 2.3 events per 1000 person-years after a primary and a booster vaccination, respectively. Male gender, older age, lower SES, a history of COVID-19, and a recent hospitalization were factors associated with a higher risk of severe COVID-19 after a primary vaccination. Our findings are consistent with those reported in other regions. For example, studies from Qatar [5], the United Kingdom [6], and the United States [7] have shown that vaccine effectiveness against SARS-CoV-2 infection declines over time, especially in older individuals and those with comorbidities. These studies similarly highlight the crucial role of booster vaccinations in mitigating the risk of severe COVID-19 outcomes among high-risk populations, supporting the trends observed in our Dutch cohort.

Only immune deficiency and obesity were independently associated with a higher risk of infection after a primary vaccination, compared with those without the respective comorbidity. Immune deficiency, diabetes mellitus, and cardiovascular, neurological, lung, or kidney disease were all associated with a higher risk of severe COVID-19 after a primary vaccination. A booster vaccination reduced the risk of severe COVID-19 in all of these comorbidities, except for persons with diabetes mellitus or lung disease, in whom the risk remained significantly elevated. While the absolute numbers indicate a reduction in severe outcomes after booster doses, the relative risk reduction may be masked by the higher incidence of breakthrough infections. These findings underscore the critical role of booster vaccinations in preventing severe outcomes.

The gender and age-specific differences in infection risk after a primary vaccination and their changes after booster doses suggest a complex interaction of immune responses and vaccine effectiveness. These findings are also reported by others and highlight the importance of considering gender and age differences in vaccine response [9,10]. In addition, the increased risk of severe COVID-19 among individuals with a history of COVID-19, despite a reduced risk of reinfection, emphasizes the role of booster doses in preventing severe outcomes in this population, especially among high-risk populations. Additionally, the association between SES and risk of breakthrough infections or severe COVID-19 highlights the importance of addressing social determinants of health in vaccination campaigns. Although we found that male gender and lower SES were associated with a lower risk of breakthrough infection, this may reflect lower testing rates or healthcare use in these groups, leading to underdetection of mild cases. These findings contrast with other studies reporting higher infection risks [25]. Furthermore, the observed inverse association with recent hospitalization is likely not causal. Rather, it may reflect a reduced exposure during inpatient isolation or a lower likelihood of community-based testing shortly after discharge, leading to underdetection of infections. This highlights the importance of interpreting such associations with caution.

Our findings are in line with research highlighting the effectiveness of booster vaccinations in reducing the risk of severe COVID-19 [26,27,28]. Moreover, our study supports evidence indicating an increased risk of severe COVID-19 in individuals with diabetes [11]. However, there is a paucity of evidence regarding the effectiveness of COVID-19 vaccines in persons with specific comorbidities. Several studies report a lower vaccine effectiveness in persons with comorbidities, compared to those without comorbidities, as opposed to investigating the effectiveness in specific comorbidities separately [10,29,30]. This is understandable, considering the complexity of these conditions, and possible limitations due to the potential scarcity of individuals with those conditions in their data. Of note, persons with comorbidities or of older age are more likely to seek medical care, potentially increasing the likelihood of infection being recorded. This may lead to an underestimation of infection rates in healthier or younger individuals and overrepresentation in groups with higher healthcare use. Nevertheless, the present study adds robust estimates of vaccine effectiveness in older persons and in those with these specific comorbidities.

To our knowledge, our study is among the first to provide evidence on the effectiveness of COVID-19 vaccines in individuals with and without specific comorbidities, addressing the previously noted lack of evidence regarding these risk factors. Consequently, the results of our study are particularly important in filling this research gap. Moreover, our findings underscore the need for risk-based vaccination programs that prioritize patients not only based on age but also on other relevant factors. Importantly, the clinical relevance of our study lies in its contribution to understanding the interplay between the investigated risk factors and SARS-CoV-2 breakthrough infection or severe COVID-19 in the context of both primary and booster vaccination. The identified factors associated with increased or decreased risk—such as age, gender, socioeconomic status, and comorbidities—provide valuable insights for targeted public health interventions.

Our findings have important clinical and public health implications. The identification of specific high-risk groups—such as older adults and individuals with comorbidities like diabetes mellitus and lung disease—highlights the need for targeted vaccination strategies. Risk-based approaches, prioritizing early booster vaccinations and possibly additional doses for these vulnerable populations, could help reduce the burden of severe COVID-19 outcomes. Furthermore, our results support the continuation of surveillance programs that monitor vaccine effectiveness across different subgroups over time, particularly as new variants emerge. These strategies are consistent with national guidelines that identify high-risk groups for COVID-19 vaccination prioritization [31]. Our study was performed with the use of a large and diverse GP cohort, representative of the Dutch population [23]. This allowed for robust analyses across multiple demographic and clinical factors. The comprehensive assessment of comorbidities provides a nuanced understanding of their influence on breakthrough infections and severe outcomes. However, limitations arising from the complex nature of the vaccination process, the urgency involved, and the allocation of vaccinations and boosters to undefined risk groups, along with potential biases inherent in observational studies, were difficult to control. The absence of information due to incompleteness of dossiers on certain variables interacted with the evolving nature of the pandemic. The emergence of new variants and time-dependent risk-shielding measures in the populations may also influence the generalizability of our findings over time. Furthermore, it is possible that persons who were in fact vaccinated were not included in our study cohorts, due to limitations resulting from the use of routine healthcare data. However, the proportion of vaccinated persons out of all active persons in our database is in line with the proportion of persons that are vaccinated as determined by the national registries [32]. Additionally, our models may be subject to residual confounding due to unmeasured factors such as ethnicity, test-seeking behavior, healthcare access, or differences in exposure risk. These factors were not available in our data, and while adjustment for SES may partly capture some of these effects, the possibility of residual bias remains. Despite these limitations, the results of our study can still be of importance in public health strategies, by describing trends of these outcomes in the vaccinated population, also addressing differences in effectiveness of vaccination in high-risk populations. While most reported associations are moderate, they remain statistically and clinically relevant, particularly in informing risk-based vaccination strategies.

## 5. Conclusions

In conclusion, our study shows that booster vaccinations play a crucial role in reducing rates of breakthrough infections and severe COVID-19, especially in high-risk populations. The results of our study suggest that a booster vaccination is most important in older persons, and those with specific comorbidities such as diabetes mellitus or lung disease. The differential impact of boosters on severe outcomes in individuals with comorbidities highlights the need for ongoing surveillance and tailored, risk-based vaccination strategies.

## Figures and Tables

**Table 1:** Patients’ characteristics at baseline, SARS-CoV-2 breakthrough infection rates, and severe COVID-19 rates, for persons receiving primary vaccination (cohort 1), and for persons receiving booster vaccination (cohort 2).

	Cohort 1			Cohort 2		
	Vaccinated N (%)	SARS-CoV-2 Infection, *n* (IR/1000 PY)	Severe COVID-19, *n* (IR/1000 PY)	Vaccinated N (%)	SARS-CoV-2 Infection, *n* (IR/1000 PY)	Severe COVID-19, *n* (IR/1000 PY)
Overall	1,090,567 (100)	12,050 (29.9)	1233 (3.1)	344,153 (100)	38,637 (256.4)	350 (2.3)
Gender						
Female	538,787 (49.4)	5558 (29.1)	719 (3.8)	163,339 (47.5)	18,323 (258.3)	223 (3.1)
Male	551,748 (50.6)	6492 (30.7)	514 (2.4)	180,810 (52.5)	20,314 (254.7)	127 (1.6)
Age						
18–59	600,257 (55.0)	7736 (39.9)	248 (1.3)	72,273 (21.0)	13,971 (459.1)	43 (1.5)
60–69	169,530 (15.5)	1633 (23.4)	247 (3.5)	97,834 (28.4)	11,445 (274.6)	99 (2.4)
70–79	153,964 (14.1)	1531 (21.0)	350 (4.8)	107,033 (31.1)	9192 (189.9)	96 (2.0)
80+	83,780 (7.7)	900 (18.1)	380 (7.6)	66,745 (19.4)	3943 (122.1)	112 (3.5)
Socioeconomic status						
Low	287,326 (26.3)	2853 (27.0)	386 (3.7)	83,968 (24.4)	7918 (223.3)	102 (2.9)
Middle	399,699 (36.7)	4447 (29.5)	492 (3.3)	126,345 (36.7)	13,094 (235.1)	129 (2.3)
High	397,653 (36.5)	4690 (32.4)	352 (2.4)	113,824 (33.1)	15,131 (296.7)	107 (2.1)
Unknown	5889 (0.5)	60 (29.2)	3 (1.5)	20,016 (5.8)	2494 (292.3)	12 (1.4)
History of COVID-19 ^1^						
No	1,049,850 (96.3)	11,891 (30.6)	993 (2.6)	331,432 (96.3)	38,002 (263.0)	235 (1.6)
Yes	40,717 (3.7)	159 (11.1)	240 (16.7)	12,721 (3.7)	635 (102.6)	115 (18.6)
Recent hospitalization ^2^						
No	1,087,104 (99.7)	12,012 (29.9)	1150 (2.9)	342,614 (99.6)	38,518 (256.7)	324 (2.2)
Yes	3463 (0.3)	38 (26.1)	83 (56.8)	1539 (0.4)	119 (176.8)	26 (38.5)
Smoking status						
Never	202,309 (18.6)	2039 (23.2)	293 (3.3)	101,577 (29.5)	8856 (199.7)	110 (2.5)
Current	77,021 (7.1)	743 (24.7)	107 (3.6)	25,901 (7.5)	2235 (194.8)	25 (2.2)
Former	164,435 (15.1)	2444 (32.5)	486 (6.5)	94,473 (27.5)	11,852 (271.8)	122 (2.8)
Unknown	646,802 (59.3)	6824 (32.5)	347 (1.7)	122,202 (35.5)	15,694 (306.1)	93 (1.8)
Timing of vaccination						
Q1 2021	67,540 (6.2)	1476 (32.0)	199 (4.3)	NA	NA	NA
Q2 2021	444,925 (40.8)	5784 (28.7)	703 (3.5)	NA	NA	NA
Q3 2021	539,708 (49.5)	4743 (31.0)	290 (1.9)	NA	NA	NA
Q4 2021	38,394 (3.5)	47 (18.6)	41 (16.2)	344,153 (100)	38,637 (256.4)	350 (2.3)
Comorbidities						
Diabetes mellitus	90,820 (8.3)	1110 (27.0)	325 (7.9)	47,966 (13.9)	4290 (198.0)	92 (4.2)
Cardiovascular disease	333,644 (30.6)	3768 (24.7)	897 (5.9)	189,299 (55.0)	18,041 (211.0)	259 (3.0)
Neurological disease	36,525 (3.3)	406 (25.1)	117 (7.2)	16,723 (4.9)	1558 (207.3)	31 (4.1)
Lung disease	192,112 (17.6)	2458 (31.2)	560 (7.1)	73,757 (21.4)	8413 (257.5)	141 (4.3)
Malignancy	125,129 (11.5)	1435 (24.4)	364 (6.2)	74,810 (21.7)	7339 (215.4)	101 (3.0)
Kidney disease	55,627 (5.1)	647 (23.6)	264 (9.6)	33,950 (9.9)	2776 (176.0)	73 (4.6)
Immune deficiency	102,940 (9.4)	1469 (31.9)	444 (9.6)	41,578 (12.1)	4786 (254.2)	95 (5.0)
Obesity	115,175 (10.6)	1567 (31.8)	282 (5.7)	52,294 (15.2)	5573 (240.6)	71 (3.1)

^1^ SARS-CoV-2 infection before first vaccination, ^2^ Hospitalization within 28 days before date of primary vaccination (cohort 1) or booster vaccination (cohort 2). NA, not applicable; IR, incidence rate; PY, person-year. Primary vaccination is defined as having received two doses of BNT162b2, mRNA-1273 or AZD1222, or one dose of Ad26.COV2.S. Booster vaccination is defined as having received a third dose, being either BNT162b2 or mRNA-1273. Persons may suffer from several combinations of comorbidities.

**Table 2:** Analyses of Poisson-adjusted incidence rate ratios of SARS-CoV-2 breakthrough infection, or severe COVID-19, for persons receiving primary vaccination, and receiving booster vaccination.

	SARS-CoV-2 Infection		Severe COVID-19	
	Primary Vaccination, IRR (95% CI)	Booster Vaccination, IRR (95% CI)	Primary Vaccination, IRR (95% CI)	Booster Vaccination, IRR (95% CI)
	N = 1,090,567	N = 344,153	N = 1,090,567	N = 344,153
Gender				
Female	1.00 (ref)	1.00 (ref)	1.00 (ref)	1.00 (ref)
Male	0.84 (0.78–0.89) ***	0.98 (0.94–1.03) ^NS^	1.48 (1.25–1.75) ***	2.00 (1.42–2.81) ***
Age				
18–59	1.00 (ref)	1.00 (ref)	1.00 (ref)	1.00 (ref)
60–69	0.78 (0.71–0.85) ***	0.62 (0.59–0.66) ***	2.94 (2.18–3.97) ***	1.56 (0.91–2.69) ^NS^
70–79	0.78 (0.72–0.86) ***	0.49 (0.47–0.52) ***	4.96 (3.80–6.47) ***	1.23 (0.71–2.13) ^NS^
80+	0.83 (0.75–0.92) ***	0.36 (0.34–0.39) ***	9.90 (7.68–12.8) ***	1.91 (1.11–3.31) *
Socioeconomic status				
Low	1.00 (ref)	1.00 (ref)	1.00 (ref)	1.00 (ref)
Middle	1.18 (1.08–1.28) ***	1.05 (0.99–1.12) ^NS^	0.92 (0.76–1.13) ^NS^	0.90 (0.59–1.37) ^NS^
High	1.20 (1.11–1.31) ***	1.33 (1.26–1.41) ***	0.67 (0.54–0.83) ***	0.88 (0.57–1.35) ^NS^
Unknown	0.93 (0.57–1.54) ^NS^	1.23 (1.11–1.35) ***	0.41 (0.07–2.26) ^NS^	0.60 (0.24–1.48) ^NS^
History of COVID-19 ^1^				
No	1.00 (ref)	1.00 (ref)	1.00 (ref)	1.00 (ref)
Yes	0.30 (0.22–0.41) ***	0.38 (0.32–0.45) ***	4.86 (3.85–6.15) ***	14.8 (10.5–20.7) ***
Recent hospitalization ^2^				
No	1.00 (ref)	1.00 (ref)	1.00 (ref)	1.00 (ref)
Yes	0.84 (0.47–1.50) ^NS^	0.71 (0.48–1.04) ^NS^	20.7 (14.9–28.8) ***	19.3 (10.5–35.3) ***
Smoking status				
Current	1.00 (ref)	1.00 (ref)	1.00 (ref)	1.00 (ref)
Former	1.62 (1.41–1.88) ***	1.51 (1.37–1.66) ***	1.94 (1.41–2.67) ***	1.27 (0.65–2.48) ^NS^
Never	1.03 (0.89–1.20) ^NS^	1.09 (0.98–1.20) ^NS^	1.01 (0.72–1.41) ^NS^	1.15 (0.58–2.26) ^NS^
Unknown	1.16 (1.01–1.33) *	1.50 (1.36–1.65) ***	0.43 (0.31–0.61) ***	0.82 (0.41–1.63) ^NS^
Timing of vaccination				
Q1 2021	1.00 (ref)	NA	1.00 (ref)	NA
Q2 2021	0.58 (0.53–0.63) ***	NA	0.59 (0.48–0.73) ***	NA
Q3 2021	0.42 (0.38–0.46) ***	NA	0.19 (0.15–0.26) ***	NA
Q4 2021	0.08 (0.02–0.23) ***	NA	0.32 (0.08–1.20) ^NS^	NA
Comorbidities				
Diabetes mellitus	0.99 (0.88–1.11) ^NS^	0.88 (0.82–0.95) ***	1.45 (1.18–1.78) ***	1.58 (1.05–2.38) *
Cardiovascular disease	0.95 (0.87–1.03) ^NS^	0.99 (0.94–1.04) ^NS^	1.73 (1.36–2.21) ***	1.54 (1.00–2.37) ^NS^
Neurological disease	1.00 (0.85–1.17) ^NS^	1.02 (0.92–1.13) ^NS^	1.48 (1.13–1.95) **	1.27 (0.68–2.37) ^NS^
Lung disease	1.04 (0.96–1.13) ^NS^	1.05 (0.99–1.11) ^NS^	1.87 (1.54–2.27) ***	1.56 (1.07–2.29) *
Malignancy	0.99 (0.90–1.09) ^NS^	1.10 (1.04–1.16) ***	1.17 (0.97–1.41) ^NS^	1.04 (0.71–1.53) ^NS^
Kidney disease	0.96 (0.84–1.10) ^NS^	1.00 (0.92–1.09) ^NS^	1.28 (1.04–1.59) *	1.42 (0.91–2.20) ^NS^
Immune deficiency	1.21 (1.10–1.34) ***	1.19 (1.11–1.27) ***	1.83 (1.50–2.24) ***	1.47 (0.96–2.25) ^NS^
Obesity	1.17 (1.06–1.29) **	0.96 (0.90–1.03) ^NS^	1.17 (0.94–1.46) ^NS^	1.07 (0.69–1.65) ^NS^

^1^ SARS-CoV-2 infection before first vaccination, ^2^ Hospitalization within 28 days before date of vaccination. IRR, incidence rate ratio; NA, not applicable. Primary vaccination is defined as having received two doses of BNT162b2, mRNA-1273 or AZD1222, or one dose of Ad26.COV2.S. Booster vaccination is defined as having received a third dose, being either BNT162b2 or mRNA-1273. IRRs for comorbidities are adjusted for all other available variables, including respective other listed comorbidities; IRRs for other variables are unadjusted. Persons may suffer from several combinations of comorbidities. *** *p*-value of <0.001; ** *p*-value of <0.01; * *p*-value of <0.05; NS: *p*-value of ≥0.05.

## Data Availability

The datasets generated and analyzed during the current study are not publicly available but are available from the corresponding author on reasonable request and after approval of the Compliance Committee of Stichting Informatievoorziening voor Zorg en Onderzoek.

## Supplementary Materials

The following supporting information can be downloaded at: https://www.mdpi.com/article/10.3390/vaccines13060564/s1, Table S1. Definitions of variables. Table S2. Sensitivity analysis of Poisson-adjusted incidence rate ratios of SARS-CoV-2 breakthrough infection for separate COVID-19 vaccines, for persons receiving primary vaccination. Table S3. Sensitivity analysis of Poisson-adjusted incidence rate ratios of SARS-CoV-2 breakthrough infection for separate COVID-19 vaccines, for persons receiving booster vaccination. Table S4. Sensitivity analysis of Poisson-adjusted incidence rate ratios of severe COVID-19 for separate COVID-19 vaccines, for persons receiving primary vaccination. Table S5. Sensitivity analyses of Poisson-adjusted incidence rate ratios of severe COVID-19 for separate COVID-19 vaccines, for persons receiving booster vaccination. Table S6. Sensitivity analyses of Poisson-adjusted incidence rate ratios of severe COVID-19, for persons receiving primary vaccination, and persons receiving booster vaccination, with all-cause mortality defined as death within 90 days of SARS-CoV-2 infection instead of 28 days. Table S7. Analyses of Poisson-adjusted incidence rate ratios of SARS-CoV-2 breakthrough infection, for persons receiving primary vaccination, and persons receiving booster vaccination. Table S8. Analyses of Poisson-adjusted incidence rate ratios of severe COVID-19, for persons receiving primary vaccination, and persons receiving booster vaccination.
