# Supplementary material for: Rates of SARS-CoV-2 Breakthrough Infection or Severe COVID-19 and Associated Risk Factors After Primary and Booster Vaccination Against COVID-19 in the Netherlands
Source: Vaccines (Basel). 2025 May 26;13(6):564. doi: 10.3390/vaccines13060564 (PMC12197751; doi:10.3390/vaccines13060564)
Supplement: Supplementary file 1 [file vaccines-13-00564-s001.zip › supplementary_information_20250506.pdf]

Supplementary Information

Supplementary Table S1. Definitions of variables.

Variable	Description	Categories	Code(s)
Age	2021 minus year of birth.	18-69, 70-79, 80+ years	NA
Gender	Gender as registered in electronic health record.	Female, male	NA
Socioeconomic status	Based on income, education and unemployment levels, on a postal code level, divided into tertiles.	Low, middle, high, unknown	NA
History of COVID-19	History of SARS-CoV-2 infection before date of second vaccine dose.	Y/N	NA
Recent hospitalization	Hospitalization within 28 days before vaccination (either dose two or booster).	Y/N	NA
Smoking status	Most recent measurement before date of vaccination (dose 2), with maximum lookback-window.	Current, former, never, unknown	WCIA 1379
Timing of vaccination	Quarter of year 2021 in which persons received the second vaccine dose.	Q1, Q2, Q3, Q4	NA
Diabetes mellitus	Yes if ICPC code is present, maximum lookback-window.	Y/N	ICPC T90
Cardiovascular disease	Yes if ICPC codes are present, maximum lookback-window.	Y/N	ICPC K73, K74, K75, K76, K77, K78, K79, K80, K82, K84, K86, K87, K89, K90, K91, K92
Neurological disease	Yes if ICPC codes are present, maximum lookback-window.	Y/N	ICPC P70, N86, N87, N88, N99
Lung disease	Yes if either ICPC, ATC or WCIA codes are present. Maximum lookback-window for ICPC, 2 years for WCIA, ATC.	Y/N	ICPC R70, R82, R89, R91, R95, R96, R99.06, T99.10, K93; WCIA 2209, 3549; ATC H02AB06, H02AB07
Malignancy	Yes if ICPC codes are present, maximum lookback-window.	Y/N	ICPC X75, X76, X77, Y78 N74, U75, U76, U77, T71, D75, D76, W72, A79, R84, R85, R86, B72, B73, B74, S77
Kidney disease	Yes if either ICPC codes are present, or when eGFR is below 30. Maximum lookback-window for ICPC, 1 year for eGFR.	Y/N	ICPC U85, U88, U99 (except U99.04); WCIA 524, 1918, 1919, 3583
Immune deficiency	Yes if either ICPC or ATC codes are present. Maximum lookback-window for ICPC, 6 months for ATC.	Y/N	ICPC B90, T99.01; ATC H02A*, L04A*
Obesity	Yes if either ICPC code is present or most recent BMI ≥ 30. Maximum lookback-window for ICPC, 1 year for BMI.	Y/N	ICPC T82, WCIA 1272

BMI, body mass index; eGFR, estimated glomerular filtration rate; ICPC, International Classification of Primary Care; WCIA, examination codes as provided by the Dutch College of General Practitioners (NHG); ATC, Anatomical Therapeutic Chemical Classification System; NA, not applicable.

Supplementary **Table S2**. Sensitivity analysis of Poisson-adjusted incidence rate ratios of SARS-CoV-2 breakthrough infection for separate COVID-19 vaccines, for persons receiving primary vaccination.

	**BNT162b2, IRR (95% CI)**	**ChAdOx1, IRR (95% CI)**	**mRNA-1273, IRR (95% CI)**	**AD26.COV2.S, IRR (95% CI)**
	**N = 792,349**	**N = 103,189**	**N = 78,755**	**N = 46,972**
Gender				
Female	1.00 (ref)	1.00 (ref)	1.00 (ref)	1.00 (ref)
Male	0.84 (0.78-0.91)***	0.65 (0.53-0.80)***	0.91 (0.65-1.28)^{NS}	0.98 (0.77-1.25)^{NS}
Age				
18-59	1.00 (ref)	1.00 (ref)	1.00 (ref)	1.00 (ref)
60-69	0.96 (0.85-1.08)^{NS}	0.35 (0.29-0.42)***	1.22 (0.46-3.24)^{NS}	0.68 (0.02-2.27)^{NS}
70-79	0.80 (0.73-0.89)***	0.47 (0.17-1.31)^{NS}	1.09 (0.25-4.73)^{NS}	0.70 (0.09-5.40)^{NS}
80+	0.86 (0.77-0.95)**	0.20 (0.04-1.13)^{NS}	1.43 (0.57-3.60)^{NS}	NA
Socioeconomic status				
Low	1.00 (ref)	1.00 (ref)	1.00 (ref)	1.00 (ref)
Middle	1.18 (1.07-1.31)***	1.15 (0.90-1.49)^{NS}	0.83 (0.54-1.27)^{NS}	1.31 (0.94-1.83)^{NS}
High	1.16 (1.05-1.29)**	1.37 (1.06-1.76)*	1.02 (0.67-1.55)^{NS}	1.37 (0.98-1.90)^{NS}
Unknown	0.75 (0.38-1.49)^{NS}	1.33 (0.40-4.38)^{NS}	1.36 (0.15-12.3)^{NS}	1.92 (0.50-7.36)^{NS}
History of COVID-19[1]				
No	1.00 (ref)	1.00 (ref)	1.00 (ref)	1.00 (ref)
Yes	0.30 (0.21-0.44)***	0.27 (0.12-0.62)***	0.42 (0.11-1.70)^{NS}	0.27 (0.07-0.98)*
Recent hospitalization[2]				
No	1.00 (ref)	1.00 (ref)	1.00 (ref)	1.00 (ref)
Yes	0.91 (0.48-1.71)^{NS}	0.56 (0.07-4.85)^{NS}	NA	0.94 (0.04-24.8)^{NS}
Smoking status				
Current	1.00 (ref)	1.00 (ref)	1.00 (ref)	1.00 (ref)
Former	1.56 (1.32-1.84)***	1.70 (1.14-2.55)^{NS}	2.48 (1.09-5.62)*	2.71 (1.11-6.65)*
Never	0.97 (0.82-1.15)^{NS}	1.05 (0.69-1.60)^{NS}	1.39 (0.62-3.12)^{NS}	1.58 (0.68-3.69)^{NS}
Unknown	1.03 (0.88-1.21)^{NS}	1.61 (1.11-2.35)*	1.19 (0.57-2.49)^{NS}	1.72 (0.83-3.58)^{NS}
Timing of vaccination				
Q1 2021	1.00 (ref)	NA	NA	NA
Q2 2021	0.55 (0.50-0.60)***	NA	NA	NA
Q3 2021	0.42 (0.38-0.47)***	NA	NA	NA
Q4 2021	0.06 (0.02-0.24)***	NA	NA	NA
Comorbidities				
Diabetes mellitus	0.98 (0.86-1.11)^{NS}	0.98 (0.69-1.39)^{NS}	1.49 (0.81-2.75)^{NS}	0.98 (0.27-3.54)^{NS}
Cardiovascular disease	0.92 (0.83-1.02)^{NS}	0.96 (0.75-1.22)^{NS}	0.85 (0.53-1.36)^{NS}	1.28 (0.78-2.09)^{NS}
Neurological disease	1.02 (0.85-1.22)^{NS}	0.82 (0.43-1.56)^{NS}	0.87 (0.34-2.17)^{NS}	0.75 (0.23-2.50)^{NS}
Lung disease	1.06 (0.97-1.17)^{NS}	1.14 (0.88-1.48)^{NS}	0.94 (0.59-1.50)^{NS}	1.10 (0.70-1.72)^{NS}
Malignancy	0.98 (0.88-1.09)^{NS}	1.16 (0.86-1.56)^{NS}	0.84 (0.42-1.68)^{NS}	0.74 (0.30-1.83)^{NS}
Kidney disease	0.92 (0.79-1.06)^{NS}	1.09 (0.64-1.84)^{NS}	1.05 (0.41-2.70)^{NS}	1.62 (0.54-4.84)^{NS}
Immune deficiency	1.19 (1.07-1.34)**	1.22 (0.89-1.65)^{NS}	1.43 (0.81-2.52)^{NS}	1.17 (0.57-2.37)^{NS}
Obesity	1.20 (1.07-1.34)**	1.07 (0.82-1.40)^{NS}	1.01 (0.57-1.82)^{NS}	1.22 (0.65-2.32)^{NS}

[1]COVID-19 infection before first vaccination, [2]Hospitalization within 28 days before date of vaccination. IRR, incidence rate ratio; NA, not applicable. Primary vaccination is defined as having received two doses of BNT162b2, mRNA-1273, ChAdOx1, or one dose of AD26.COV2.S. IRRs for comorbidities are adjusted for all other available variables, including respective other listed comorbidities; IRRs for other variables are unadjusted. ***p-value of <0.001; **p-value of <0.01; *p-value of <0.05; NS: p-value of ≥0.05.

Supplementary **Table S3**. Sensitivity analysis of Poisson-adjusted incidence rate ratios of SARS-CoV-2 breakthrough infection for separate COVID-19 vaccines, for persons receiving booster vaccination.

	**BNT162b2, IRR (95% CI)**	**mRNA-1273, IRR (95% CI)**
	**N = 67,958**	**N = 191,222**
Gender		
Female	1.00 (ref)	1.00 (ref)
Male	0.81 (0.74-0.89)***	1.08 (1.03-1.14)*
Age		
18-59	1.00 (ref)	1.00 (ref)
60-69	0.53 (0.48-0.60)***	0.73 (0.68-0.79)***
70-79	0.35 (0.31-0.41)***	0.57 (0.53-0.62)***
80+	0.28 (0.24-0.32)***	0.39 (0.36-0.43)***
Socioeconomic status		
Low	1.00 (ref)	1.00 (ref)
Middle	1.04 (0.92-1.17)^{NS}	1.08 (1.00-1.16)^{NS}
High	1.28 (1.13-1.44)***	1.23 (1.15-1.33)***
Unknown	0.78 (0.62-0.98)*	1.40 (1.24-1.57)***
History of COVID-19[1]		
No	1.00 (ref)	1.00 (ref)
Yes	0.43 (0.32-0.57)***	0.28 (0.23-0.35)***
Recent hospitalization[2]		
No	1.00 (ref)	1.00 (ref)
Yes	0.51 (0.24-1.10)^{NS}	0.86 (0.53-1.38)^{NS}
Smoking status		
Current	1.00 (ref)	1.00 (ref)
Former	1.32 (1.08-1.61)**	1.41 (1.25-1.59)***
Never	0.90 (0.73-1.10)^{NS}	1.36 (1.21-1.54)***
Unknown	1.51 (1.26-1.82)***	1.48 (1.31-1.67)***
Comorbidities		
Diabetes mellitus	0.85 (0.72-1.00)^{NS}	0.92 (0.84-1.00)*
Cardiovascular disease	0.85 (0.75-0.95)**	1.03 (0.97-1.10)^{NS}
Neurological disease	0.90 (0.71-1.13)^{NS}	1.05 (0.92-1.19)^{NS}
Lung disease	1.02 (0.91-1.14)^{NS}	1.09 (1.02-1.17)**
Malignancy	1.01 (0.89-1.15)^{NS}	1.10 (1.04-1.18)**
Kidney disease	0.94 (0.77-1.15)^{NS}	1.01 (0.92-1.11)^{NS}
Immune deficiency	1.19 (1.03-1.37)*	1.20 (1.11-1.30)***
Obesity	1.01 (0.88-1.16)^{NS}	0.96 (0.89-1.04)^{NS}

[1]COVID-19 infection before first vaccination, [2]Hospitalization within 28 days before date of vaccination. IRR, incidence rate ratio. Booster vaccination is defined as having received a third dose, being either BNT162b2 or mRNA-1273. IRRs for comorbidities are adjusted for all other variables, including respective other listed comorbidities; IRRs for other variables are unadjusted. ***p-value of <0.001; **p-value of <0.01; *p-value of <0.05; NS: p-value of ≥ 0.05.

Supplementary **Table S4**. Sensitivity analysis of Poisson-adjusted incidence rate ratios of severe COVID-19 for separate COVID-19 vaccines, for persons receiving primary vaccination.

	**BNT162b2, IRR (95% CI)**	**ChAdOx1, IRR (95% CI)**	**mRNA-1273, IRR (95% CI)**	**AD26.COV2.S, IRR (95% CI)**
	**N = 792,349**	**N = 103,189**	**N = 78,755**	**N = 46,972**
Gender				
Female	1.00 (ref)	1.00 (ref)	1.00 (ref)	NA
Male	1.49 (1.23-1.81)***	2.16 (1.25-3.74)**	0.79 (0.35-1.78)^{NS}	NA
Age				
18-59	1.00 (ref)	1.00 (ref)	1.00 (ref)	NA
60-69	2.37 (1.56-3.61)***	2.02 (0.92-4.45)^{NS}	5.49 (1.16-26.0)*	NA
70-79	4.70 (3.47-6.37)***	8.52 (2.00-36.3)**	10.3 (2.04-51.7)**	NA
80+	9.23 (6.90-12.3)***	4.43 (0.56-34.8)^{NS}	24.2 (9.80-59.5)***	NA
Socioeconomic status				
Low	1.00 (ref)	1.00 (ref)	1.00 (ref)	NA
Middle	0.92 (0.73-1.15)^{NS}	0.71 (0.37-1.34)^{NS}	0.57 (0.20-1.63)^{NS}	NA
High	0.61 (0.48-0.78)***	0.74 (0.39-1.44)^{NS}	0.90 (0.35-2.32)^{NS}	NA
Unknown	0.20 (0.01-3.41)^{NS}	0.93 (0.04-23.4)^{NS}	NA	NA
History of COVID-19[1]				
No	1.00 (ref)	1.00 (ref)	1.00 (ref)	NA
Yes	4.32 (3.26-5.71)***	4.25 (2.13-8.47)***	13.2 (5.5-31.4)***	NA
Recent hospitalization[2]				
No	1.00 (ref)	1.00 (ref)	1.00 (ref)	NA
Yes	20.0 (13.9-28.8)***	10.3 (2.46-43.2)**	34.7 (7.9-153)***	NA
Smoking status				
Current	1.00 (ref)	1.00 (ref)	1.00 (ref)	NA
Former	2.05 (1.41-2.97)***	1.08 (0.47-2.49)^{NS}	1.59 (0.42-6.01)^{NS}	NA
Never	1.04 (0.71-1.54)^{NS}	0.71 (0.30-1.72)^{NS}	0.78 (0.21-2.94)^{NS}	NA
Unknown	0.45 (0.30-0.66)***	0.49 (0.21-1.14)^{NS}	0.27 (0.08-0.96)*	NA
Timing of vaccination				
Q1 2021	1.00 (ref)	NA	NA	NA
Q2 2021	0.70 (0.56-0.87)**	NA	NA	NA
Q3 2021	0.18 (0.13-0.25)***	NA	NA	NA
Q4 2021	0.28 (0.06-1.34)^{NS}	NA	NA	NA
Comorbidities				
Diabetes mellitus	1.42 (1.14-1.79)**	1.47 (0.74-2.93)^{NS}	1.35 (0.44-4.16)^{NS}	NA
Cardiovascular disease	1.61 (1.21-2.13)***	1.80 (0.95-3.40)^{NS}	1.81 (0.62-5.27)^{NS}	NA
Neurological disease	1.58 (1.18-2.12)**	1.48 (0.47-4.64)^{NS}	2.75 (0.04-1.86)^{NS}	NA
Lung disease	2.05 (1.65-2.55)***	1.24 (0.66-2.33)^{NS}	1.58 (0.58-4.30)^{NS}	NA
Malignancy	1.08 (0.87-1.33)^{NS}	2.30 (1.27-4.16)**	0.80 (0.27-2.43)^{NS}	NA
Kidney disease	1.29 (1.02-1.63)*	1.77 (0.76-4.13)^{NS}	1.75 (0.56-5.43)^{NS}	NA
Immune deficiency	1.69 (1.35-2.11)***	2.41 (1.27-4.56)**	2.62 (0.94-7.28)^{NS}	NA
Obesity	1.15 (0.90-1.47)^{NS}	1.28 (0.67-2.47)^{NS}	0.73 (0.22-2.45)^{NS}	NA

[1]COVID-19 infection before first vaccination, [2]Hospitalization within 28 days before date of vaccination. IRR, incidence rate ratio; NA, not applicable. Primary vaccination is defined as having received two doses of BNT162b2, mRNA-1273, ChAdOx1, or one dose of AD26.COV2.S. IRRs for comorbidities are adjusted for all other available variables, including respective other listed comorbidities; IRRs for other variables are unadjusted. ***p-value of <0.001; **p-value of <0.01; *p-value of <0.05; NS: p-value of ≥0.05.

Supplementary **Table S5**. Sensitivity analyses of Poisson-adjusted incidence rate ratios of severe COVID-19 for separate COVID-19 vaccines, for persons receiving booster vaccination.

	**BNT162b2, IRR (95% CI)**	**mRNA-1273, IRR (95% CI)**
	**N = 67,958**	**N = 191,222**
Gender		
Female	1.00 (ref)	1.00 (ref)
Male	1.89 (1.01-3.54)*	2.30 (1.41-3.78)***
Age		
18-59	1.00 (ref)	1.00 (ref)
60-69	2.62 (1.00-6.86)^{NS}	1.33 (0.59-2.97)^{NS}
70-79	3.53 (1.36-9.14)**	0.82 (0.36-1.89)^{NS}
80+	2.91 (1.09-7.80)*	1.74 (0.79-3.81)^{NS}
Socioeconomic status		
Low	1.00 (ref)	1.00 (ref)
Middle	0.76 (0.37-1.58)^{NS}	1.11 (0.57-2.16)^{NS}
High	0.50 (0.21-1.20)^{NS}	1.34 (0.71-2.55)^{NS}
Unknown	0.50 (0.10-2.39)^{NS}	0.95 (0.28-3.16)^{NS}
History of COVID-19[1]		
No	1.00 (ref)	1.00 (ref)
Yes	16.4 (8.84-30.5)***	15.4 (9.65-24.5)***
Recent hospitalization[2]		
No	1.00 (ref)	1.00 (ref)
Yes	14.2 (4.65-43.2)***	20.4 (8.1-51.1)***
Smoking status		
Current	1.00 (ref)	1.00 (ref)
Former	1.42 (0.44-4.55)^{NS}	1.13 (0.47-2.74)^{NS}
Never	0.85 (0.26-2.80)^{NS}	0.94 (0.37-2.38)^{NS}
Unknown	0.60 (0.18-1.96)^{NS}	0.67 (0.26-1.72)^{NS}
Comorbidities		
Diabetes mellitus	1.76 (0.82-3.78)^{NS}	1.35 (0.76-2.41)^{NS}
Cardiovascular disease	1.72 (0.73-4.03)^{NS}	1.07 (0.60-1.92)^{NS}
Neurological disease	1.58 (0.57-4.33)^{NS}	1.14 (0.45-2.86)^{NS}
Lung disease	1.81 (0.90-3.64)^{NS}	1.37 (0.79-2.36)^{NS}
Malignancy	1.45 (0.73-2.89)^{NS}	0.91 (0.52-1.58)^{NS}
Kidney disease	0.80 (0.32-2.00)^{NS}	1.90 (1.06-3.43)^{NS}
Immune deficiency	1.41 (0.66-3.00)^{NS}	1.30 (0.69-2.44)^{NS}
Obesity	1.00 (0.43-2.32)^{NS}	1.06 (0.57-1.97)^{NS}

[1]COVID-19 infection before first vaccination, [2]Hospitalization within 28 days before date of vaccination. IRR, incidence rate ratio. Booster vaccination is defined as having received a third dose, being either BNT162b2 or mRNA-1273. IRRs for comorbidities are adjusted for all other available variables, including respective other listed comorbidities; IRRs for other variables are unadjusted. ***p-value of <0.001; **p-value of <0.01; *p-value of <0.05; NS: p-value of ≥0.05.

Supplementary **Table S6**. Sensitivity analyses of Poisson-adjusted incidence rate ratios of severe COVID-19, for persons receiving primary vaccination, and persons receiving booster vaccination, with all-cause mortality defined as death within 90 days of SARS-CoV-2 infection instead of 28 days.

	**Primary vaccination, IRR (95% CI)**	**Booster vaccination, IRR (95% CI)**
Gender		
Female	1.00 (ref)	1.00 (ref)
Male	1.46 (1.25-1.71)***	1.92 (1.39-2.67)***
Age		
18-59	1.00 (ref)	1.00 (ref)
60-69	3.07 (2.30-4.08)***	1.60 (0.93-2.73)^{NS}
70-79	5.14 (3.80-6.63)***	1.33 (0.77-2.29)^{NS}
80+	11.2 (8.80-14.2)***	2.23 (1.31-3.79)**
Socioeconomic status		
Low	1.00 (ref)	1.00 (ref)
Middle	0.91 (0.76-1.10)^{NS}	0.93 (0.62-1.40)^{NS}
High	0.69 (0.56-0.85)***	0.92 (0.61-1.39)^{NS}
Unknown	0.53 (0.13-2.16)^{NS}	0.60 (0.25-1.44)^{NS}
History of COVID-19		
No	1.00 (ref)	1.00 (ref)
Yes	4.13 (3.27-5.21)***	12.9 (9.30-17.9)***
Recent hospitalization		
No	1.00 (ref)	1.00 (ref)
Yes	18.0 (12.9-24.9)***	17.5 (9.54-32.0)***
Smoking status		
Current	1.00 (ref)	1.00 (ref)
Former	1.91 (1.42-2.57)***	1.31 (0.68-2.51)^{NS}
Never	0.99 (0.72-1.36)^{NS}	1.20 (0.62-2.32)^{NS}
Unknown	0.43 (0.31-0.58)***	0.86 (0.44-1.68)^{NS}
Timing of vaccination		
Q1 2021	1.00 (ref)	NA
Q2 2021	0.57 (0.48-0.69)***	NA
Q3 2021	0.18 (0.14-0.23)***	NA
Q4 2021	0.28 (0.08-1.03)^{NS}	NA
Comorbidities		
Diabetes mellitus	1.46 (1.21-1.77)***	1.60 (1.08-2.36)*
Cardiovascular disease	1.76 (1.40-2.21)***	1.65 (1.08-2.52)*
Neurological disease	1.66 (1.30-2.11)***	1.46 (0.83-2.55)^{NS}
Lung disease	1.80 (1.50-2.16)***	1.47 (1.02-2.12)*
Malignancy	1.23 (1.03-1.46)*	1.16 (0.81-1.66)^{NS}
Kidney disease	1.42 (1.17-1.72)***	1.49 (0.99-2.25)^{NS}
Immune deficiency	1.90 (1.57-2.28)***	1.58 (1.05-2.36)*
Obesity	1.12 (0.92-1.39)^{NS}	1.03 (0.67-1.57)^{NS}

[1]COVID-19 infection before first vaccination, [2]Hospitalization within 28 days before date of vaccination. IRR, incidence rate ratio; NA, not applicable. primary vaccination is defined as having received two doses of BNT162b2, mRNA-1273, ChAdOx1, or one dose of AD26.COV2.S. Booster vaccination is defined as having received a third dose, being either BNT162b2 or mRNA-1273. IRRs for comorbidities are adjusted for all other variables, including respective other listed comorbidities; IRRs for other variables are unadjusted. ***p-value of <0.001; **p-value of <0.01; *p-value of <0.05; NS: p-value of ≥0.05.

Supplementary **Table S7.** Analyses of Poisson-adjusted incidence rate ratios of SARS-CoV-2 breakthrough infection, for persons receiving primary vaccination, and persons receiving booster vaccination.

	**Primary vaccination, IRR (95% CI)**			**Booster vaccination, IRR (95% CI)**		
**Comorbidities**	**Model 1**	**Model 2**	**Model 3**	**Model 1**	**Model 2**	**Model 3**
Diabetes mellitus	1.13 (1.02-1.26)	1.01 (0.91-1.13)	0.99 (0.88-1.11)	0.94 (0.88-1.01)	0.88 (0.82-0.94)	0.88 (0.82-0.95)
Cardiovascular disease	1.08 (1.00-1.17)	0.96 (0.89-1.05)	0.95 (0.87-1.03)	1.04 (0.99-1.09)	0.99 (0.94-1.04)	0.99 (0.94-1.04)
Neurological disease	1.04 (0.89-1.23)	1.00 (0.85-1.17)	1.00 (0.85-1.17)	1.02 (0.92-1.14)	1.03 (0.92-1.14)	1.02 (0.92-1.13)
Lung disease	1.19 (1.10-1.28)	1.10 (1.02-1.19)	1.04 (0.96-1.13)	1.12 (1.06-1.17)	1.10 (1.05-1.16)	1.05 (0.99-1.11)
Malignancy	1.04 (0.95-1.15)	1.01 (0.91-1.11)	0.99 (0.90-1.09)	1.12 (1.06-1.19)	1.11 (1.05-1.18)	1.10 (1.04-1.16)
Kidney disease	1.07 (0.94-1.21)	0.97 (0.85-1.11)	0.96 (0.84-1.10)	1.02 (0.95-1.11)	1.00 (0.93-1.09)	1.00 (0.92-1.09)
Immune deficiency	1.30 (1.18-1.43)	1.23 (1.12-1.36)	1.21 (1.10-1.34)	1.22 (1.14-1.30)	1.22 (1.14-1.30)	1.19 (1.11-1.27)
Obesity	1.26 (1.15-1.38)	1.16 (1.06-1.29)	1.17 (1.06-1.29)	1.00 (0.94-1.06)	0.94 (0.88-1.00)	0.96 (0.90-1.03)

IRR, incidence rate ratio; NA, not applicable. Primary vaccination is defined as having received two doses of BNT162b2, mRNA-1273 or ChAdOx1, or one dose of AD26.COV2.S. Booster vaccination is defined as having received a third dose, being either BNT162b2 or mRNA-1273. In model 1 we adjusted for age and gender. In model 2 we used model 1 and additionally adjusted for socioeconomic status, history of SARS-CoV-2 infection, recent hospitalization, smoking status, and timing of vaccination. In model 3, we used model 2 and additionally adjusted for all other comorbidities, other than the one that was investigated.

Supplementary **Table S8.** Analyses of Poisson-adjusted incidence rate ratios of severe COVID-19, for persons receiving primary vaccination, and persons receiving booster vaccination.

	**Primary vaccination, IRR (95% CI)**			**Booster vaccination, IRR (95% CI)**		
**Comorbidities**	**Model 1**	**Model 2**	**Model 3**	**Model 1**	**Model 2**	**Model 3**
Diabetes mellitus	1.78 (1.46-2.16)	1.58 (1.29-1.93)	1.45 (1.18-1.78)	1.86 (1.27-2.72)	1.69 (1.14-2.51)	1.58 (1.05-2.38)
Cardiovascular disease	2.09 (1.67-2.61)	1.97 (1.55-2.50)	1.73 (1.36-2.21)	1.80 (1.21-2.67)	1.68 (1.10-2.58)	1.54 (1.00-2.37)
Neurological disease	1.58 (1.20-2.08)	1.51 (1.15-1.99)	1.48 (1.13-1.95)	1.43 (0.77-2.67)	1.31 (0.71-2.45)	1.27 (0.68-2.37)
Lung disease	2.76 (2.32-3.27)	2.49 (2.09-2.96)	1.87 (1.54-2.27)	2.10 (1.50-2.96)	1.84 (1.30-2.60)	1.56 (1.07-2.29)
Malignancy	1.36 (1.13-1.64)	1.29 (1.07-1.56)	1.17 (0.97-1.41)	1.18 (0.81-1.74)	1.10 (0.75-1.62)	1.04 (0.71-1.53)
Kidney disease	1.74 (1.41-2.15)	1.57 (1.26-1.94)	1.28 (1.04-1.59)	1.83 (1.18-2.83)	1.65 (1.07-2.57)	1.42 (0.91-2.20)
Immune deficiency	2.89 (2.42-3.45)	2.58 (2.16-3.09)	1.83 (1.50-2.24)	2.23 (1.52-3.27)	1.88 (1.27-2.77)	1.47 (0.96-2.25)
Obesity	1.61 (1.31-1.97)	1.42 (1.15-1.76)	1.17 (0.94-1.46)	1.48 (0.99-2.22)	1.32 (0.86-2.01)	1.07 (0.69-1.65)

IRR, incidence rate ratio; NA, not applicable. Primary vaccination is defined as having received two doses of BNT162b2, mRNA-1273 or ChAdOx1, or one dose of AD26.COV2.S. Booster vaccination is defined as having received a third dose, being either BNT162b2 or mRNA-1273. In model 1 we adjusted for age and gender. In model 2 we used model 1 and additionally adjusted for socioeconomic status, history of SARS-CoV-2 infection, recent hospitalization, smoking status, and timing of vaccination. In model 3, we used model 2 and additionally adjusted for all other comorbidities, other than the one that was investigated.
